# Tuberculosis, an Infectious Disease

**Published:** 1880-07-20

**Authors:** 


### TUBERCULOSIS AN INFECTIOUS DISEASE.

We have at last some daylight let in upon this most obscure field in nosology. Tuberculosis will henceforth take place among the infectious diseases. Tuberculosis is a sort of key-note in the whole gamut of internal diseases, and the most far-sighted pathologists and clinicians have always expended the greater part of their lives in its elucidation. Tuberculosis runs into every department of medicine, into internal medicine, into surgery, into obstetrics and gynecology; it is the key-stone in the arch of pathology. Physicians, surgeons, obstetricians, every one engaged in the science or art of medicine will hail with pleasure anything definite, anything settled in the history of tuberculosis. The transmissibility of tuberculosis has been hinted at long ago, but it has never until now received the sanction of such authority as to establish its acceptance. No one will dispute the accuracy of Cohnheim's investigations or the validity of his conclusions. We present our readers a full abstract to-day of the remarkably lucid and comprehensive report made by Cohnheim to the Leipsic faculty a few months ago.

The report marks an epoch in the history of this disease which in its practical significance far exceeds in value the works of Lænnec and Virchow. It is taken from the Medizin. Neuigk f. prakt. Aerzte, April 3, 1880:

The most important advance in our knowledge of tuberculosis was contributed by Villemin's discovery of the transmissibility of this disease. Although the discovery was doubted for a long time, by Cohnheim as well as by others, it must now be looked upon as a fully established fact, as proven by the following investigation of Cohnheim and Solomonsen:

If the smallest particle of tuberculous matter be carried through a lineal incision of the cornea into the aqueous chamber of the eye of a rabbit, there appears, after a period of incubation of about six weeks, an eruption upon the iris of minute nodules, which increase to a certain size and then undergo caseous degeneration, to be followed in the course of months by a more or less general tuberculosis of the lungs, peritoneum and various other organs.

Of the greatest significance is the fact that this result occurs regularly, but only when real tuberculous matter has been inoculated. We may therefore utilize this inoculability as a diagnostic criterion of tuberculous products, a fact which is so much the more important in that the anatomico-morphological character of tuberculosis does not suffice in all cases to differentiate this affection with certainty from syphilitic products on the one hand, and on the other from other non-specific, but simply chronic irritative conditions. Neither the nodular form, the histological structure, the occurrence of giant cells, caseation, nor all these circumstances together are absolutely characteristic of tuberculosis. The only absolutely perfect and certain criterion is the capacity and infection.

And taking this criterion as a stand we must include as tuberculous, caseous pneumonia, the so-called caseation of lymph glands, as well as as the fungous joint inflammations in most cases, while simple elements like lupus tissues not being inoculable are not tuberculous.

As carriers of the infection of tuberculosis we assume parasitic specific organisms, though it is true that we are not as yet able to demonstrate them with certainty.

The tuberculous virus reaehes the body in the great majority of cases through the inspired air. Thus arises first tuberculosis of the lungs; which may then develop tuberculosis of the pleura, of the bronchial glands and of the great air passages. In some rare cases, the tuberculosis may originate in the larynx. Later the virus is carried by the sputa into the alimentary canal. Thence develops the so frequent clasic picture of pulmonary-intestinal tuberculosis.

On the other hand, the virus may first enter the digestive canal, an occurrence observed most frequently in children and dependent upon the ingestion of milk from tuberculous cows, that is of cows suffering from the fecal disease. Thus arises the *phthisis mescraica.* It is probable also that the so-called tuberculous inflammations of the mouth and larynx, as well as the caseous swellings of the cervical lymph glands arise in this way.

According to reports of Weigert tuberculous menengitis may then follow in certain cases from a migration of the tuberculous poison into the cranium from the upper nasal cavities. But urogenital tuberculosis is to be regarded as produced through excretion. The tuberculous virus, like other corpuscular elements, penetrates the glomeruli, having passed from the blood into the urinary passages and then develops its effects along these preformed canals.

In cases of primary bone and joint tuberculosis which are mostly referred to traumatic causes, it must be assumed that the poison is already in the blood and is simply extravasated by the trauma in greater quantity in the affected regions.

To account for the rapidity and universality of the dissemination of tuberculous products in the cases of acute generalized tuberculosis we must invoke a profuse inundation of the juicy organs with tuberculous virus. This explanation is rendered probable by the anatomical findings of tuberculosis of the thoracic duct and of the pulmonary veins in such cases.

On the other hand there are many cases, as is well known, of purely localized tuberculosis, in which the disease is found limited to a definite locality. But there is no essential difference in these cases. For the local limitation of the process is either explicable by the short duration of the disease (as in the local tuberculosis of the aged) or, where it has existed long, it is still in process of extension, though the process is slow. But it is perfectly certain that tuberculosis may result in perfect recovery. That the so-called local tuberculosis represents nothing essentially different is proven above all things by the fact that its products are just as inoculable as are those of general tuberculosis. The relation between local and general tuberculosis is very much like that between chancre and constitutional syphilis. A chancre, whether soft or hard, may induce a general infection of the body, may do it, but not must do it. Even so it is with local tuberculosis.

For the rest individual predispositions come into play. In the experimental induction of tuberculosis, differences manifest themselves with regard to the extension of the process and the manner of the extension.

But so far as regards the *phthisical habitus*, it has nothing to do with the susceptibility to tuberculosis. Such individuals are already tuberculous, and are tuberculous mostly by heredity. Tuberculous virus can pass into the products of generation, into the semen and ovum. The disease is thus present in the new-born child, but may break out only after a lateral stage of many years, just like hereditary syphilis in which, however, the latest stage is usually shorter. But during this latest stage the virus present in the body so effects the development of the body as to give rise to phthisical habits.

So, in tuberculosis, everything depends on the virus. Such a thing as predisposition to tuberculosis is false. We discover at all points the closest analogies between tuberculosis and syphilis. Both require, above all things, infection, transmissibility of the disease from person to person.—*J. T. W. in Lancet & Clinic.*

